# Protothecal peritonitis in child after bone marrow transplantation: case report and literature review of paediatric cases

**DOI:** 10.1002/nmi2.61

**Published:** 2014-09-14

**Authors:** T Sykora, J Horakova, D Buzzasyova, M Sladekova, M Poczova, S Sufliarska

**Affiliations:** 1 Department of Paediatric Haematology and Oncology, Haematopoietic Stem Cell Transplantation Unit, Comenius University Children's HospitalBratislava, Slovakia; 2 Department of Paediatric Anaesthesiology and Intensive Medicine, Comenius University Children's HospitalBratislava, Slovakia; 3 Department of Mycology, HPL Ltd.Bratislava, Slovakia

**Keywords:** Bone marrow transplantation, child, peritoneal dialysate, *Prototheca wickerhamii*, protothecosis

## Abstract

The case presented here illustrates a protothecal infection caused by *Prototheca wickerhamii* in a paediatric haematopoietic stem cell recipient followed by a review of the literature of all 13 paediatric cases published since 1980. Protothecosis is a rare disease caused by algae, not described in this setting before. Infection was proven additionally post-mortem from peritoneal dialysis fluid. Even though no death of a paediatric patient due to this infection has been reported and the mortality rate associated with protothecosis is low, our patient died from multiorgan failure as a result of numerous post-transplant complications and a strain of cultivated alga that was highly resistant to antifungal agents. *Prototheca* spp. show various susceptibility profiles, and there is no direct correlation between *in vitro* activity and clinical response. There are different treatment regimens described but there are no clear published guidelines of specific therapy of protothecosis. Paediatric cases were successfully treated mostly with amphotericin B and azoles. As the number of immunocompromised patients increases, it is necessary to think more about unusual pathogens such as *Prototheca*.

## Case Report

A 3-year-old boy with Philadelphia-chromosome-positive, high-risk acute lymphoblastic leukaemia achieved the first complete remission and underwent allogeneic stem cell transplantation (SCT) from a 10/10 HLA-matched unrelated donor. His conditioning regimen consisted of Fludarabine/Treosulphan/Thiotepa and anti-thymocyte globulin + cyclosporin A as a graft-versus-host disease (GVHD) prophylaxis. The graft was infused after erythrodepletion due to ABO incompatibility.

**Figure 1:**
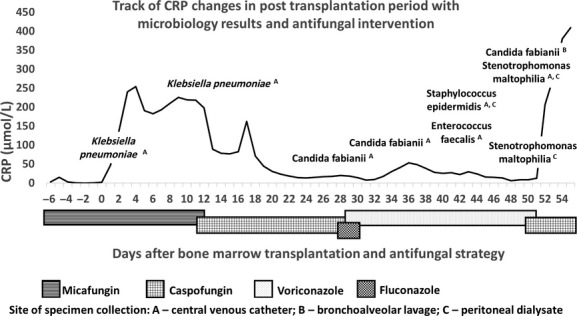
Course of present case with C reactive protein changes, microbiology findings and antifungal therapy.

Day +1 after SCT he developed febrile neutropenia. Aetiology of this febrile episode was later identified from a blood culture as *Klebsiella pneumoniae* and it was treated according to the antibiotic sensitivity tests. In the peri-transplant period he developed a severe veno-occlusive liver disease with high bilirubin blood level, body fluid retention and ascites with the necessity for abdominal drain insertion. Later, anuria and respiratory failure developed and the patient was transferred to the intensive care unit for mechanical ventilation and peritoneal dialysis. Bilirubin blood levels continued to rise up to 857 μmol/L (50.1 mg/dL), a molecular adsorbent recirculation system was used four times. On neutrophil engraftment day +21 the patient's condition had improved—laboratory inflammation markers were decreased as well as fever. On day +26, the intestinal form of GVHD developed with massive intestinal bleeding. *Candida fabianii* and multiresistant *Staphylococcus epidermidis* and *Enterococcus faecalis* were identified as microbial agents causing other concomitant infections (Fig.1).

Despite 39 days of peritoneal dialysis, the kidney function of the patient did not improve and another febrile episode developed with increased laboratory inflammation markers. Blood cultures and dialysate culture showed multiresistant *Stenotrophomonas maltophilia* to be the cause of sepsis. Despite intensive and multidisciplinary care the patient died on day +55 after SCT. In addition, the result of dialysate culture revealed a very rare *Prototheca wickerhamii*. Autopsy was not performed.

## Discussion

*Prototheca* spp. are ubiquitous chlorophyllous algae belonging to the Chlorophyceae. Besides various environmental niches, *Prototheca* spp. have been found colonizing the human skin, fingernails and respiratory and digestive systems 1. It is not a common hospital-borne infection. Hospital-acquired cases have been associated with surgery and orthopaedic procedures, but human-to-human transmission has not been reported 1. The source of the infection in this case is unknown.

**Figure 2:**
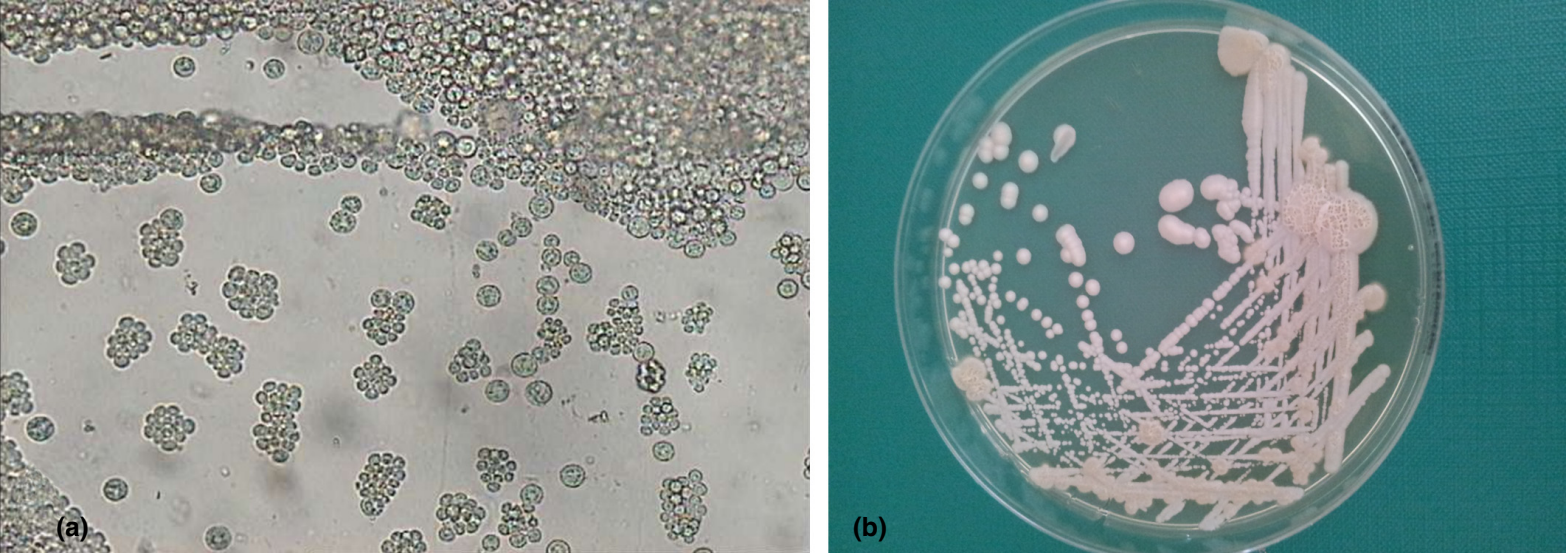
Microscopic and cultivation results. (a) Direct microscopy of large cells resembling yeast-like formation without budding (× 200 magnification). (b) White and pale cream-coloured yeast-like colonies on Sabouraud dextrose agar.

Five species of *Prototheca* have been distinguished, of which only two—*P. wickerhamii* and *Prototheca zopfii*—are described as pathogenic in humans 1. In the present case white and pale cream-coloured yeast-like colonies were grown on Sabouraud dextrose agar with antibiotics after 5 days of incubation at 30°C. Direct microscopy showed large Gram-positive cells of various sizes resembling yeast-like formations but budding was absent (Fig.2). Further micromorphological and biochemical description was made with identification of the strain as *P. wickerhamii*.

According to animal experiments, *Prototheca* spp. seem to have low virulence, overall their pathogenicity is moderate and protothecosis is considered a rare opportunistic infection 2,3.

According to the clinical presentation, there are three distinguished clinical forms known—cutaneous infection, bursitis and systemic infection. The most common are cutaneous infection and olecranon bursitis; systemic dissemination or organ involvement represents only 10% of all reported infections 4. Previous review studies suggested a 2.2% attributable mortality rate, which represents a lower number compared with candidaemia, but individual outcome still depends on the history and clinical context of each patient 5. Among all patients with cancer and protothecosis, overall mortality was 54% and attributable mortality was 14% 6.

Patients who are immunocompromised due to steroid use or who have underlying haematological/solid malignancy or AIDS have a higher risk of protothecosis.

More than 120 cases of protothecosis have been reported and it is believed that a much greater number of cases are unreported, perhaps because of morphological confusion with other microbes such as *Lacazia lobii*, *Coccidio desimmitis*, *Histoplasma duboisii*, *Blastomyces dermatitis* or *Pneumocystis jirovecii* 7.

Protothecosis in children mostly presents as infections involving various organs. From the 13 cases reported five were cutaneous, with or without olecranon bursitis, one was a catheter-related infection and the rest had other organ involvement (Table1). Generally, *Prototheca* spp. show various susceptibility profiles, and there is no direct correlation between *in vitro* activity and clinical response 1. There are no published guidelines of specific treatments for protothecosis. Algae are susceptible to amphotericin B and most of them were resistant to 5-flucytosine, fluconazole and itraconazole 5. In contrast to this, voriconazole shows superior activity against *P. wickerhamii* 1. In the case described here, the *P. wickerhamii* strain was fully susceptible only to amphotericin B, voriconazole was not effective (Table2). Paediatric cases were successfully treated mostly by amphotericin B and azoles (Table1). From review papers it can generally be concluded that most failures are associated with monotherapy by azoles 1. The therapeutic response of paediatric patients to amphotericin B treatment is very good, even though the optimal dosage and duration of an antifungal therapy are unknown. The only death of a child with protothecosis reported is our present patient. Antifungal treatment and prophylaxis of our patient followed the SCT guidelines using micafungin antimycotic prophylaxis. Changes of anti-infective agents followed the recommendations of the European Society for Blood and Marrow Transplantation guidelines according to the current clinical status and microbial finding in the patient (Fig.1). However, the anti-infective management was very challenging because of the kidney failure and other post-transplantation complications.

**Table 1:** Review of 13 paediatric cases of protothecosis published since 1980

Patient no.	Age/sex	Medical history	Site/organ involvement	Diagnosis	Treatment	Outcome	Reference
1	7 years/M	Hodgkin's lymphoma	Catheter tip	Culture *P. wickerhamii*	Catheter removal Am B	Cure	Leimann *et al*., 2004 (Heney *et al*., 1991)
2	17 years/F	Surgery, ganglion removal	Hand abscess	Culture *P. wickerhamii*	Multiple excisions	Cure	Leimann *et al*., 2004 (Iacoviello *et al*., 1992 Holcomb, 1981^a^)
3	8 months/?	Unlisted	Gastroenteritis	Culture *P. wickerhamii*	None	Unlisted	Leimann *et al*., 2004 (Iacoviello *et al*., 1992 Casal, 1983^a^)
4	6 years/F	Unlisted	Vulva	Culture *P. wickerhamii*	Gentian violet, steroids	Cure	Leimann *et al*., 2004 (Iacoviello *et al*., 1992 Nelson, 1987^a^)
5	5 years/F	Unlisted	Upper lip	Culture *P. wickerhamii*	Ketoconazole	Good response	Leimann *et al*., 2004 (Iacoviello *et al*., 1992 Kuo, 1987^a^)
6	15 years/M	Unlisted	Small intestine, liver	Histopathology and culture *P. wickerhamii*	Am B + fluconazole	Unlisted	Leimann *et al*., 2004 (Ravisse *et al*., 1993 Matsuda, 1991^b^)
7	13 years/M	Anaemia	Small intestine + lymph nodes	Histopathology and culture *P. wickerhamii*	Am B	Unlisted	Leimann *et al*., 2004 (Ravisse *et al*., 1993 Matsuda, 1991^b^)
8	78 days/M	Very low birthweight	Endocarditis	Histopathology and culture *P. wickerhamii*	Resection of atrium mass Am B	Cure	Leimann *et al*., 2004 (Buendra *et al*., 1999)
9	10 years/M	Combined immunodeficiency	Skin + olecranon bursitis	Culture *P. wickerhamii*	Am B + itraconazole IVIG	Good response	Mathew *et al*., 2010
10	6 months/M	Congenital hydrocephalus	Central nervous system	Microscopy and molecular identification	Ketoconazole Fluconazole + Am B	Cure	Zak *et al*., 2012
11	14 years/M	Unlisted	Skin	Microscopy and culture *Prototheca* spp.	Itraconazole	Cure	Kalsy *et al*., 2012
12	4 years/F	Liver transplantation, immunosuppression	Lungs	Culture *P. wickerhamii*	Am B	Cure	Tan *et al*., 2013
13	2 years/F	Submental and foot abscess	Skin	Microscopy identification	Am B + gentamicin Itraconazole	Cure	Tello–Zavala *et al*., 2013
14	3 years/M	ALL Ph+, MUD BMT, multiorgan failure	Peritoneal dialysate	Culture *P. wickerhamii*	None	Death	Here presented

References from Table1: Leimann *et al*., 2004 4; Heney *et al*., 1991 8; Iacoviello *et al*., 1992 9; Ravisse *et al*., 1993 10; Buendra *et al*., 1998 11; Mathew *et al*., 2010 12; Zak *et al*., 2012 13; Kalsy *et al*., 2012 14; Tan *et al*., 2013 15; Tello-Zavala *et al*., 2013 16. Am B, amphotericin B; ALL Ph+, positive high-risk acute lymphoblastic leukaemia; IVIG, intravenous immunoglobulin; MUD BMT, unrelated matched donor bone marrow transplantation.

a Cases mentioned and referred to in Iacoviello's review (1992).

b Cases mentioned and referred to in Ravisse's review (1993).

**Table 2:** Minimal inhibition concentration profile of *Prototheca wickerhamii* strain cultivated in the present case

Antifungal agent	Dosage (mg/L)	Susceptibility
Voriconazole	32.0	Resistant
Posaconazole	2.0	Susceptible only to higher dosage
Amphotericin B	0.094	Susceptible
Fluconazole	256.0	Resistant
Itraconazole	32.0	Resistant
Echinocandins (micafungin, anidulafungin, caspofungin)	32.0	Resistant

Protothecal infection usually runs with other co-pathogens such as cytomegalovirus, herpes simplex virus, *Staphyloccocus aureus*, *Enterococcus faecalis*, *Pseudomonas aeruginosa*, *Klebsiella pneumoniae*, *Escherichia coli*, *Cryptococcus* spp., *Candida glabrata* and *Aspergillus* spp. 1. In the present case different co-pathogens were identified: Gram-negative bacterium *Stenotrophomonas maltophilia* and *Candida fabianii* (Fig.1). Cytomegalovirus, Epstein–Barr virus, adenovirus and BK virus infections repeatedly tested negative on PCR. Besides viruses, mannan, anti-mannan and galactomannan antigen testing was run for complete mycology follow up. Microbiological tests were performed routinely every 3–4 days; collection of specimens was performed in sterile conditions. Infection developed on caspofungin, meropenem and acyclovir. Amphotericin B appears to be the most effective drug for systemic protothecosis, although it has been reported to be ineffective in some cases. So far it is recommended as the first-line therapy in cases of dissemination and for patients who are severely immunocompromised, or have other underlying illness 17. This recommendation was irrelevant for our patient because of the kidney failure that did not improve.

Duration of microbial identification in our settings is approximately 4 days. The results of the cultures from the peritoneal dialysate were obtained after the patient had died. It is evident that the unstable and critical condition of the patient played a key role in his death and the protothecal infection was only a contributory factor. Regarding the source of infection, the reported data of protothecosis in patients with long-lasting peritoneal dialysis showed that the catheter was infected. In the case described here, the peritoneal catheter was replaced 1–2 days before death, due to its malfunction and clogging with blood coagula. We have no evidence of contaminated surgical equipment during the handling procedure. Previous peritoneal dialysates tested negative; this was the only positive specimen. After the identification of *P. wickerhamii*, a sample of the alga was deposited in the mycology laboratory archives.

According to published data, this is the first reported case of protothecosis in a paediatric bone marrow transplantation recipient. There is a rising probability of infection by less common and more atypical infectious organisms. *Prototheca* is a rare pathogen and so is not usually suspected. As the number of immunocompromised patients increases, it is necessary to think more of unusual pathogens when it comes to diagnosis of infectious complications during treatment.
